# Influence of hypomagnetic field on the heartbeat in zebrafish embryos

**DOI:** 10.3389/fphys.2022.1040083

**Published:** 2022-10-21

**Authors:** Viacheslav Krylov, Alexander Machikhin, Daniil Sizov, Anastasia Guryleva, Anastasia Sizova, Svetlana Zhdanova, Vladimir Tchougounov, Alexander Burlakov

**Affiliations:** ^1^ Scientific and Technological Center of Unique Instrumentation, Russian Academy of Sciences, Moscow, Russia; ^2^ Papanin Institute for Biology of Inland Waters, Russian Academy of Sciences, Borok, Russia; ^3^ Lomonosov Moscow State University, Moscow, Russia

**Keywords:** danio rerio, heartbeat rate, heart rate variability, circadian rhythm, abnormal phenotype, survival

## Abstract

The magnetic environment may influence the functioning of the cardiovascular system. It was reported that low-frequency and static magnetic fields affect hemodynamics, heart rate, and heart rate variability in animals and humans. Moreover, recent data suggest that magnetic fields affect the circadian rhythms of physiological processes. The influence of the magnetic environment on heart functionating during early development has been studied insufficiently. We utilized transparent zebrafish embryos to evaluate the effect of the hypomagnetic field on the characteristics of cardiac function using a noninvasive optical approach based on photoplethysmographic microscopic imaging. The embryos were exposed to the geomagnetic and hypomagnetic fields from the second to the 116th hour post fertilization under a 16 h light/8 h dark cycle or constant illumination. The exposure of embryos to the hypomagnetic field in both lighting modes led to increased embryo mortality, the appearance of abnormal phenotypes, and a significant increase in the embryo’s heartbeat rate. The difference between maximal and minimal heartbeat intervals, maximal to minimal heartbeat intervals ratio, and the coefficient of variation of heartbeat rate were increased in the embryos exposed to the hypomagnetic field under constant illumination from 96 to 116 h post fertilization. The dynamics of heartbeat rate changes followed a circadian pattern in all studied groups except zebrafish exposed to the hypomagnetic field under constant illumination. The results demonstrate the importance of natural magnetic background for the early development of zebrafish. The possible mechanisms of observed effects are discussed.

## Introduction

A natural magnetic background environs all living beings on the Earth. The geomagnetic field (GMF) makes the most significant contribution to the natural magnetic environment. The GMF intensity varies from about 25 to 65 μT (Alken et al., 2021). In addition, relatively weak natural magnetic fluctuations arise due to the interaction between the Earth’s magnetosphere and solar wind (Akasofu and Chapman, 1972). Biological evolution has taken place against the background of GMF, and this environmental parameter can affect physiological processes. Researchers are weakening the GMF to close to zero values in the experiments to evaluate the significance of the natural magnetic background for an organism. The biological effects of this hypomagnetic field (HMF) are compared with the control located in the unmodified GMF.

HMF is reported to affect various endpoints in different species (see, for example, reviews by Mo et al., 2012a; Binhi and Prato, 2017; Zhang and Tian, 2020; Xue et al., 2021; Zhang et al., 2021). However, little is known about its influence on cardiovascular functioning. A few studies have indicated that the exposure of adult humans to HMF leads to decreased heart rate and increased heart rate variability (Gurfinkel et al., 2016; Demin et al., 2021). Long-term monitoring of cardiac activity indicated a relationship between heart rate variability and weak natural geomagnetic variations in rabbits (Chibisov et al., 2004; Gmitrov and Gmitrova, 2004), rats (Kuzmenko et al., 2019), and humans (Chernouss et al., 2001; Janashia et al., 2022).

It is known that magnetic influences, including HMF, can significantly affect the circadian rhythms of physiological processes (Touitou et al., 2010; Xue et al., 2021). The heart rate has a pronounced circadian rhythm (Zhang et al., 2020). For example, the heart rate in larval zebrafish increases in the light and decreases in the dark phase (Fong et al., 2021). Zebrafish heart contains its circadian pacemaker at the gene expression level (Whitmore et al., 2000), and the daily change in heartbeat rate remains in excised larval hearts *in vitro* (Fong et al., 2021). Some works describe the effect of magnetic influences on circadian patterns in the rhythms of cardiac function (Makarov, 1997; Chibisov et al., 2004; McCraty et al., 2017).

We could not find publications on the influence of HMF on heart rate, heart rate variability, and circadian patterns of daily heart rate dynamics in zebrafish, which are one of the most popular vertebrate model organisms. This study aimed to evaluate the above endpoints in *Danio rerio* embryos exposed to HMF and GMF under different lighting modes.

Nowadays, *D. rerio* is used for modeling cardiovascular pathologies due to the presence of human disease genes in the genome (Howe et al., 2013). Transparent zebrafish embryos and larvae are the most convenient for heart functioning registration and vessel map reconstruction with optical approaches (Ling et al., 2022). The zebrafish’s heart and vascular anatomy are similar to that of other vertebrates, including humans (Isogai et al., 2001; Liu et al., 2016; Bowley et al., 2022). Mentioned advantages allow utilizing zebrafish embryos and larvae as a model for examining congenital heart defects (Liang et al., 2010), cardiomyopathy (Norton et al., 2011; Zou et al., 2015; Brodehl et al., 2019), and the effects of various treatments on the functioning of the cardiovascular system (Denvir et al., 2008; Santoso et al., 2019; Meng et al., 2020). In this regard, the evaluation of the HMF effects on the zebrafish’s cardiovascular functioning can reveal the significance of the natural magnetic background for the heart functioning in this model species. It also will allow one to evaluate the possible influence of the laboratory environment, which weakens or significantly modifies the magnetic background, on the results of the zebrafish cardiovascular system studying.

## Materials and methods

### Fish husbandry

Wild-type zebrafish (AB strain) were obtained from the commercial distributer and maintained in the Papanin Institute for Biology of Inland Waters. The housing conditions and maintenance procedures corresponded to the standard protocol (Avdesh et al., 2012). We kept zebrafish in glass aquaria at 26°C in a 16 h light/8 h dark cycle as preferable for zebrafish cultivation (Abdollahpour et al., 2020). All methods were carried out following relevant guidelines and regulations. The study was approved by the Institutional Animal Care and Use Committee at the Papanin Institute for Biology of Inland Waters (protocol 6, date of approval: 25/02/2022 https://ibiw.ru/index.php?p=downloads&id=46958).

#### Magnetic fields

We used two following magnetic conditions in the experiments. The first is the GMF with 51.7 μT intensity and deflection of lines of force for 72.05° from the horizontal plane Earth’s. The second is the HMF of about 0 ± 0.1 µT.

We used a coil system consisting of three pairs of mutually orthogonal Helmholtz coils (700 turns of 0.2 mm copper wire in each coil, diameter 0.5 m) made by the Schmidt Institute of Physics of the Earth (www.ifz.ru) and three (for each coil pair) sources of direct current (AKIP-1103, Manson Engineering Ltd., China) for the generation of HMF. Measurements of the vertical and horizontal components of the GMF in meridional, latitudinal, and vertical directions were taken before the experiments using a three-component fluxgate magnetometer NV0302A (ENT, Saint Petersburg, Russia). Afterward, the system of Helmholtz coils was placed so that its axes would coincide with the measured components of GMF. The current was supplied to the winding of each Helmholtz coil so that the generated magnetic field was equal in strength, but opposite in direction to the measured components of GMF. A homogeneous HMF was generated at the center of the Helmholtz coils in a cylindrical volume of 10 cm in diameter and 5 cm high. The induction of the magnetic field in any direction within this region was 0 ± 10 nT. The magnetic induction increased outside this cylindrical volume, reaching tens of microteslas near the coils. The industrial alternating magnetic fields of 50 Hz were less than 15 nT and did not appear in the harmonics.

The parameters of HMF in the Helmholtz coils and local magnetic fields (0–500 Hz) in the laboratory were checked twice a day with an NV0599C magnetometer (ENT, Saint Petersburg, Russia).

### Handling and experimental protocol

We carried out two separate experiments. The exposure of zebrafish embryos to HMF and GMF under a 16 h light/8 h dark cycle was performed during the first experiment from 30/05/2022 to 12/06/2022. The same exposures under constant illumination were carried out during the second experiment from 13/05/2022 to 25/06/2022.

The main environmental circadian light-dark zeitgeber has presented in the first experiment. The second experiment assumed the rhythm of the endogenous circadian oscillator with the absence of the light-dark external zeitgeber. We did not use constant-dark conditions to avoid that short exposure to light during the imaging could cause a phase shift or clock resetting (Tamai et al., 2007). Embryonic development is reflected in hours post fertilization (hpf).

The relatively low temperature of 23°C was maintained during the experiments to slow down the zebrafish embryonic development rate (Urushibata et al., 2021). It allows us to perform prolonged registration of heartbeats in predominantly sedentary embryos from 48 to 116 hpf. Otherwise, the zebrafish become mobile several hours after hatching, and one should use anesthesia to achieve appropriate imaging. We did not use an anesthetic in this experiment as it can affect heart rate (Craig et al., 2006).

After breeding, 400 fertilized eggs were raised and moved into two glass containers filled with 2 L of water with methylene blue (200 eggs per container). One container with developing embryos was transferred into the Helmholtz coils (HMF), and another remained in the unmodified GMF. Zebrafish in the glass containers were utilized for counting embryo mortality and abnormal phenotypes. Eggs and prelarvae in the containers were counted daily. After the hatching, developmental abnormalities were evaluated together with embryo counting.

Additionally, four eggs were used for imaging within each replication. They were placed into transparent plastic 40 mm dishes filled with water, one egg per dish. Two dishes with developing embryos were transferred into the Helmholtz coils (HMF), and the other two dishes remained in the unmodified GMF. Image data were captured every 4 h from 48 to 116 hpf (six times a day at 9:00, 13:00, 17:00, 21:00, 01:00, and 05:00). The number of dishes within one replication was limited by four to achieve precise imaging at a predetermined time of day. We performed the imaging in four independent replications for both experiments, and eight different specimens were used for further analysis.

### Image acquisition and processing

We put the dish in the light microscope Olympus CX-35 equipped with a monochrome CMOS camera TheImagingSource DMK 33UX250, centered a zebrafish heart in the field of view, and acquired a series of N = 2000 images (12 bit, 1080 × 1920 pixels) of a beating heart at 100 fps for each embryo. These stacks were processed by a well-established video capillaroscopy algorithm described by (Machikhin et al., 2020).

**FIGURE 1:**
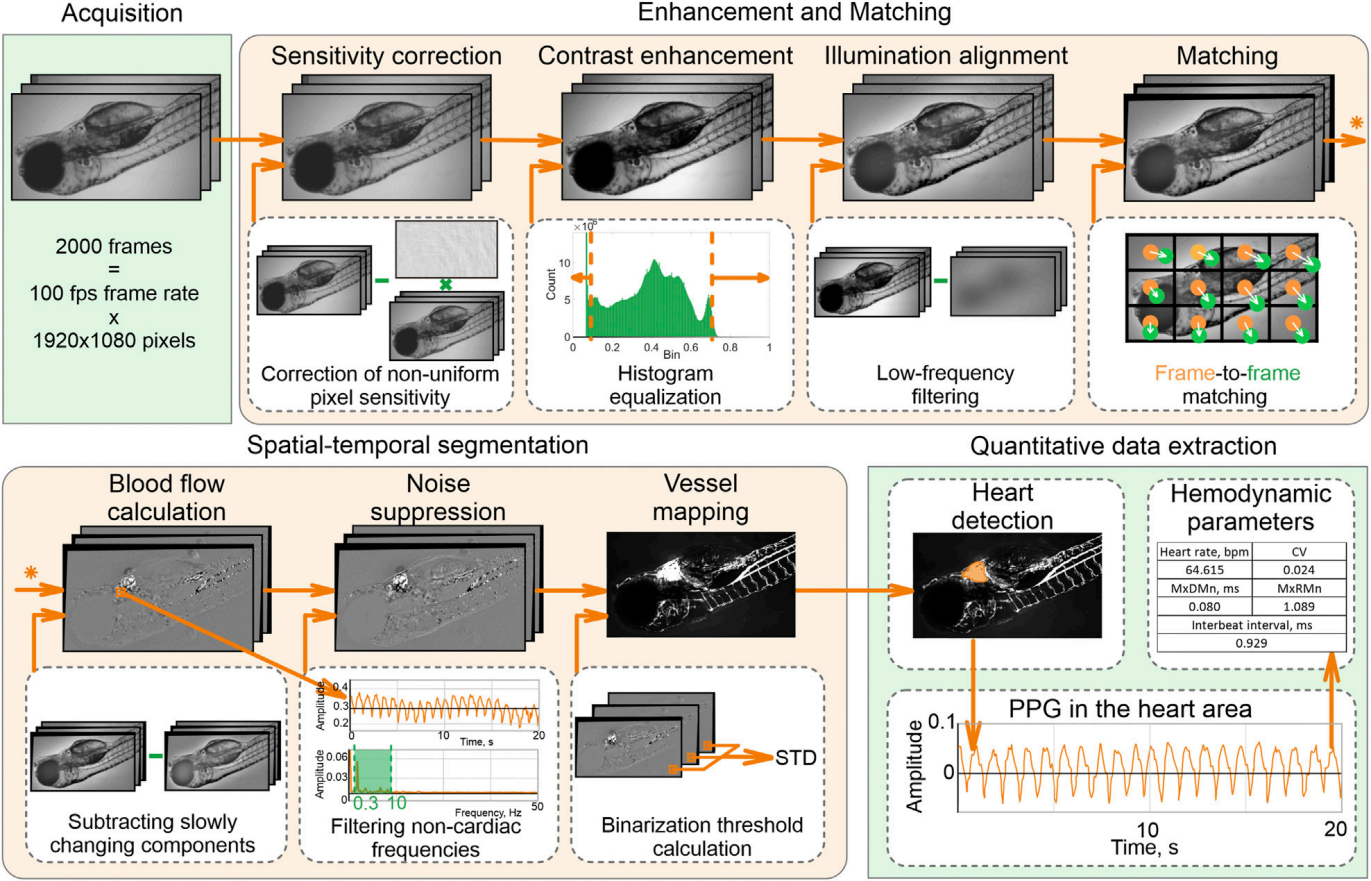
Data processing pipeline.

Our processing pipeline (Figure 1) includes image enhancement and matching, spatial-temporal segmentation, and quantification of the heart rhythm. First, we corrected the pixel sensitivity non-uniformity in all images of the stack and enhanced their contrast by adjusting the histograms to the [0,1] range. Next, we aligned spatial illumination non-uniformity by subtracting the low-frequency components. Due to anesthesia-free protocol and inevitable specimen motion, images in the stack are mismatched by both local and global shifts. We applied the GeFolki algorithm (Plyer et al., 2015) based on optical flow estimation to eliminate it.

Spatial-temporal analysis of the enhanced and well-matched image stack allows accurate detection of the heart and vessel areas. To distinguish these areas from the blood-free background, we subtracted slowly changing components, eliminated noise, and selected pixels only with pulsatile intensities related to cardiac activity. A vessel map was calculated as a standard deviation of the filtered image stack within each pixel.

The largest area in the vessel image is the heart area. We calculated a photoplethysmogram (PPG) by averaging temporal intensity dependency in this area and analyzed its shape to calculate hemodynamic parameters: heartbeat rate (beats per min), mean interbeat interval (ms), the difference between maximal and minimal heartbeat intervals (MxDMn, ms), maximal to minimal heartbeat intervals ratio (MxRMn), coefficient variation of heartbeat rate (CV).

### Statistical analysis

Survival curves were compared using a Log-rank test. Heartbeat rate and related data were tested for normality (Shapiro–Wilk W-test or Kolmogorov–Smirnov test) and homogeneity of variance (Levene’s test). As all data had a normal distribution, differences between mean values within each experiment were tested using a *t*-test. Daily changes in heartbeat rate were analyzed using a Cosinor analysis to determine if the variations have a circadian rhythm (Cornelissen, 2014). Due to the complexity of heartbeat rate dynamics from 48 to 72 hpf, the time series obtained from 72 to 116 hpf were used for the Cosinor analysis. The linear trend was subtracted from the time series.

## Results

**FIGURE 2:**
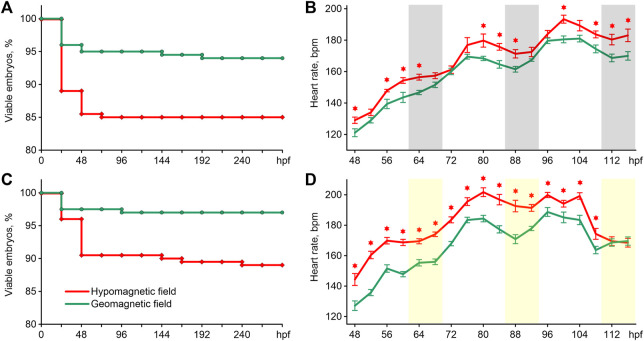
Survival curves **(A)** and an average heartbeat rate **(B)** in zebrafish exposed to hypomagnetic field and the geomagnetic field in the first experiment, and survival curves **(C)** and an average heartbeat rate **(D)** in zebrafish exposed to HMF and the geomagnetic field in the second experiment. Error bars represent standard errors. The asterisks indicate significant differences (t-test, p < 0.05). Gray bars represent the dark phase, and the light bars are the expected dark phase under constant illumination. Hpf–hours post fertilization; bpm–beats per minute.

The survival of zebrafish is shown in Figures 2A,C. The exposure of embryos to HMF in both lighting modes led to increased embryo mortality compared to the survival curve of zebrafish maintained in GMF (Log-rank test chi-square = 8.15, *p* < 0.01 for 16 h light/8 h dark cycle; chi-square = 9.41, *p* < 0.01 for constant illumination). In addition, abnormal phenotypes appeared among the embryos exposed to HMF in both lightning modes. Examples of such abnormally developing embryos are provided in supplementary videos. The percentage of abnormal phenotypes by 288 hpf was 5.5% and 12.5% of the initial number of eggs in zebrafish exposed to HMF under 16 h light/8 h dark cycle and constant illumination correspondingly. There were no abnormal phenotypes in the groups exposed to GMF.

The pattern of changes in heartbeat rate from 48 to 116 hpf had a clear trend toward an increase in frequency in all groups (Figures 2B,D). For this reason, the differences in cardiac performance were presented and assessed within three 24 h intervals (Table 1). The most pronounced effect of HMF is a significant increase in the embryo’s heartbeat rate in comparison with the exposure to GMF within each 24-h interval in both lighting modes (Table 1). The dynamics of changes in heartbeat rate under 16 h light/8 h dark cycle in the groups exposed to GMF and HMF have close values during the transition from the dark to light phase at 68–72 and 92–96 hpf. Then, during the light phase and at the beginning of the dark phase, the heartbeat rate significantly increased in embryos maintained in HMF compared to the control (Figure 2C). Under the constant illumination, the heartbeat rate in the groups exposed to HMF was significantly higher than that in GMF at all time points, except for 112 and 116 hpf.

**TABLE 1:** Parameters of cardiac activity in zebrafish exposed to hypomagnetic field (HMF) and geomagnetic field (GMF) under different light conditions. Data are given as mean ± standard error (*n* = 48, sum of the measurements registered within six time points for the 48–68 hpf, 72–92 hpf, and 96–116 hpf periods). Significant differences between groups exposed to HMF and GMF are indicated with asterisks (*p < 0.05; **p < 0.01; ***p < 0.001).

Exposure	Heart rate, bpm	Interbeat interval, ms	MxDMn, ms	MxRMn	CV
Experiment 1, 16 h light/8 h dark cycle, 48–68 hpf					
GMF	138.554 ± 1.777**	0.423 ± 0.005	0.040 ± 0.004	1.061 ± 0.005	0.018 ± 0.001
HMF	146.520 ± 1.760	0.410 ± 0.005	0.039 ± 0.003	1.065 ± 0.005	0.020 ± 0.001
Experiment 1, 16 h light/8 h dark cycle, 72–92 hpf					
GMF	165.226 ± 0.840***	0.362 ± 0.004	0.023 ± 0.005	1.054 ± 0.011	0.017 ± 0.003
HMF	172.890 ± 1.539	0.348 ± 0.008	0.032 ± 0.003	1.066 ± 0.006	0.019 ± 0.001
Experiment 1, 16 h light/8 h dark cycle, 96–116 hpf					
GMF	175.693 ± 1.147***	0.343 ± 0.004*	0.035 ± 0.003	1.085 ± 0.007	0.022 ± 0.001
HMF	185.584 ± 1.237	0.326 ± 0.004	0.032 ± 0.002	1.076 ± 0.006	0.021 ± 0.001
Experiment 2, constant light, 48–72 hpf					
GMF	145.596 ± 1.783***	0.416 ± 0.006***	0.036 ± 0.003	1.091 ± 0.008	0.020 ± 0.001
HMF	164.473 ± 1.716	0.364 ± 0.004	0.029 ± 0.002	1.080 ± 0.007	0.019 ± 0.001
Experiment 2, constant light, 72–96 hpf					
GMF	176.952 ± 1.190***	0.340 ± 0.002***	0.033 ± 0.002	1.101 ± 0.008	0.023 ± 0.001
HMF	193.598 ± 1.376	0.311 ± 0.002	0.029 ± 0.002	1.101 ± 0.007	0.024 ± 0.001
Experiment 2, constant light, 96–116 hpf					
GMF	176.531 ± 1.778**	0.342 ± 0.003*	0.036 ± 0.002**	1.111 ± 0.006**	0.025 ± 0.001**
HMF	184.242 ± 2.245	0.325 ± 0.005	0.045 ± 0.002	1.144 ± 0.007	0.030 ± 0.001

The mean interbeat interval strongly correlated with the heartbeat rate, and the changes in these characteristics are close to each other (Table 1). MxDMn, MxRMn, and the coefficient of variation reflect heart rate variability that is not dependent on heartbeat rate. No significant differences were found between the groups exposed to different magnetic conditions under studied illumination from 48 to 96 hpf in these characteristics. There were no differences in MxDMn, MxRMn, and the coefficient of variation between zebrafish maintained in HMF and GMF from 96 to 116 hpf under 16 h light/8 h dark cycle. However, the embryos exposed to the HMF under constant illumination from 96 to 116 hpf had a more variable heart rate in comparison with the zebrafish maintained in GMF (Table 1).

The dynamics of changes in heartbeat rate (Figures 2B,D) and individual changes of this indicator in both experiments (Figure Suppl. 1) show the presence of a circadian rhythm. This rhythm looks more pronounced in zebrafish maintained in a 16 h light/8 h dark cycle (Figure 2B, Figure Suppl. 1). The results of the cosinor analysis reveal the existence of a circadian rhythm in these groups with a high level of significance (Table 2). At the same time, the zero-amplitude test showed the presence of a circadian rhythm close to the threshold of significance p-level in zebrafish exposed to GMF under constant illumination. An insignificant circadian rhythm of heartbeat rate was observed in embryos kept in HMF under constant illumination. Regardless of the lighting regime, cosinor analysis showed a shift in the acrophase in zebrafish exposed to the HMF 1.3–2.3 h later relative to those zebrafish that developed in 16 h light/8 h dark cycle (Table 2).

**TABLE 2:** Cosinor analysis results of the zebrafish heart rate at different magnetic and light conditions (24 h rhythm for 72–116 hpf).

Exposure	Mesor	Amplitude	Acrophase (hours, decimal)	Zero-amplitude test (*F*, *p*-value)
Geomagnetic field, 16 h light/8 h dark	170.460	6.634	13.962	8.790, 0.008
Hypomagnetic field, 16 h light/8 h dark	179.237	7.318	15.215	10.234, 0.005
Geomagnetic field, constant light	176.741	7.876	13.331	4.286, 0.049
Hypomagnetic field, constant light	188.920	6.558	15.621	1.530, 0.268

## Discussion

The increase in embryo mortality and the appearance of abnormal phenotypes in response to the hypomagnetic influence is a substantial adverse effect. Similar effects of HMF on the early development of other species are represented in the available literature. Increased mortality in embryos of cyprinid fish roach (*Rutilus rutilus*) due to exposure to HMF was described (Krylov et al., 2021). The same effect has been revealed in mice (Fesenko et al., 2010) and rats (Korolyov et al., 2009) maintained under GMF shielding during pregnancy. Exposure of migratory planthoppers (*Laodelphax striatellus* and *Nilaparvata lugens*) to HMF decreased female fecundity and reduced the vitellogenin transcript levels of newly molted females in both planthopper species (Wan et al., 2014). A 5-day exposure of Japanese newt (*Cynops pyrrhogaster*) embryos to HMF caused somatic abnormalities and retarded development (Asashima et al., 1991). Aberrant phenotypes were distinguishable after the exposure of African clawed frog (*Xenopus laevis*) embryos to HMF (Mo et al., 2012b). The incubation of Japanese quail (*Coturnix coturnix japonica*) eggs under HMF led to embryo malformations, including abnormalities in the formation of the cardiovascular system (Trukhanov et al., 2014). The increased mortality of embryos accompanied by the appearance of abnormal phenotypes in zebrafish exposed to HMF in the present experiments is consistent with the results obtained in other species.

We could not find data on the effect of HMF on heart rate in zebrafish. Few data have been published on the influence of this factor on humans. Thus, Gurfinkel et al. (2014; 2016) report a decrease in heart rate in adults under 1–1.5 h exposure to HMF. Several studies describe the influence of changes in the magnetic background on fish species. The exposure of sea trout (*Salmo trutta*) embryos to a static magnetic field of 4 mT caused a smooth increase in the heart rate and following return to the initial level by the 30th minute of exposure. European whitefish (*Coregonus lavaretus*) embryos showed a slight and short-term reducing and following a distinct increase of the heart rate in response to the same 4 mT magnetic field (Formicki et al., 2021). The exposure of northern pike (*Esox lucius*) embryos to the 4 mT static magnetic field and common carp (*Cyprinus carpio*) embryos to 51–70 mT static magnetic fields led to similar effects as follows. The heart rate increased from the first minute of exposure, reached the maximal value by the fifth minute, and then decreased to the initial level (Winnicki et al., 1994; Formicki and Winnicki, 1996). Exposure of sea trout and European whitefish embryos to an alternating magnetic field (15 mT, 50 Hz) also caused an increase in the heart rate. However, in contrast to the effects of static fields, there was no return to the initial level, and an increased heart rate was maintained until the end of exposure (Formicki et al., 2021). Thus, the increase in the zebrafish heartbeat rate in response to HMF in the present experiment is consistent with the reactions in other fish species embryos to changes in the magnetic environment. It should be noted that both the HMF and short-term changes in the magnetic background during the transfer from HMF to the microscope for imaging could affect heart rate. However, both cases prove the importance of the natural geomagnetic environment for the functioning of the heart muscle of zebrafish embryos.

A lot of data on the effects of various chemical agents added to water on the zebrafish heart rate have been published to date. Often, exposures to toxicants lead to a decrease in heart rate (Barreto et al., 2022; Cui et al., 2022; Wang et al., 2022; Yu et al., 2022). Increased heart rate in response to the addition of chemicals to water was also described (Shabnam and Philip, 2018; Li et al., 2019; Wang et al., 2022). It is suggested that an increase or decrease in heart rate under the influence of toxicants can arise in a hormesis dose-dependent manner (Calabrese et al., 2021). In other words, the physiologically harmless doses can activate and enhance non-specific and specific adaptive mechanisms, including an increase in heart rate in fish embryos, while toxicants at significant doses cause a set of adverse effects, including a decrease in the zebrafish’s heart rate (Agathokleous, 2022). Following this, it can be assumed that the HMF effect on zebrafish is not harmful enough to cause a decrease in heart rate and leads to a compensatory increase in this rate as the low hormetic-zone influence (Agathokleous, 2022).

Changes in heart rate variability in response to the alterations in the magnetic environment are described in the literature. For example, 6-week exposure of albino Wistar rat males to electromagnetic field radiation from a dual transceiver mobile phone led to a decrease in maximal to minimal heartbeat intervals ratio (Usman et al., 2020). It has also been reported that geomagnetic storms can cause changes in human heart rate variability (Chernouss et al., 2001; Janashia et al., 2022). Slow changes in the vertical component of GMF in the millihertz range led to an increase in heart rate variability in male volunteers (Vasin et al., 2019). In our experiment, an increase in heart rate variability was observed at the interval of 96–116 hpf in the group of zebrafish exposed to HMF under constant illumination. That is, embryos required a relatively long exposure to HMF and consistent lighting for 4 days for the effect appearance. Most likely, an increase in heart rate variability in response to HMF depends on the duration of continuous exposure. However, this assumption requires further verification.

The changes in the heart functioning in those zebrafish that were maintained in HMF could be associated with the revealed increased mortality and developmental abnormality of embryos. The cardiac effects could be due to minor inconspicuous developmental disturbances in the survived embryos or the possible selective mortality of fish with a slower heart rate in HMF.

Patterns of circadian rhythm in the zebrafish heartbeat rate obtained in this experiment are consistent with the available data (Fong et al., 2021). Some studies described the influence of magnetic and electromagnetic fields on molecular oscillators and circadian rhythms in other species (Manzella et al., 2015; Agliassa and Maffei, 2019; Bartos et al., 2019). Moreover, there is a possibility that zebrafish use slow magnetic fluctuations as a secondary zeitgeber for biological circadian rhythms (Krylov et al., 2022). Therefore, the circadian rhythm disruptions under HMF and constant illumination were not unexpected. Most likely, cryptochromes are involved in the effects of magnetic fields on circadian rhythms. These proteins are crucial elements of transcription-translation negative feedback loops (Patke et al., 2020) and, at the same time, are thought to be a possible biological magnetodetector (Hore and Mouritsen, 2016). A single paper reported that exposure of zebrafish fibroblast cells to strong electromagnetic fields for 1 h over 4 days caused an increase in *Cry1aa* expression and the shifting of *Cry1aa* oscillations phase. Similarly, the exposure of larvae to the same magnetic fields between 11–14 days post fertilization led to desynchronization of *Cry1aa* circadian oscillations (Oliva et al., 2019). Further experiments are needed to verify the relationship between changes in the magnetic background and disturbances in circadian rhythms in the heart rate of zebrafish.

Several possible primary targets for magnetic fields in living cells have been proposed. Often-discussed hypotheses consider the magnetic impact on the magnetic nanoparticles in living tissues (Walker, 2008), the precession of magnetic moment (Belova and Pancheliuga, 2010), and the rate of reactions that involve spin-correlated radical pairs (Hore and Mouritsen, 2016). The last mentioned includes the cryptochrome-mediated effects of magnetic fields. Almost all suggested mechanisms emphasize the crucial role of GMF (as a static external magnetic field) for the occurrence of biological effects induced by other magnetic fields since the constant geomagnetic component determines the parameters of effective magnetic influence on molecular or submolecular targets. The absence of magnetic fields should affect all the particles with a magnetic moment that constitute living tissues due to the Zeeman effect, which consists of the splitting of a spectral line into several components in the presence of a static magnetic field (Binhi and Prato, 2017). These processes most likely underlie the effects observed in the present study. Further research is needed to elucidate pathways from primary targets to changes in cardiac activity.

This study demonstrates the importance of natural magnetic background for zebrafish’s early development. The results reveal the responses of zebrafish cardiac activity during early development to a decrease in the induction of GMF. A possible reduction of GMF caused by electrical laboratory appliances or magnetic-shielding materials should be taken into account in the studies that suggest recording zebrafish heartbeats. Given the growing popularity of *D. rerio*, accounting for magnetic conditions may improve the purity of experiments and increase the repeatability of results for a heart rate between laboratories.

## Data Availability

The original contributions presented in the study are included in the article/supplementary material, further inquiries can be directed to the corresponding author.
