# Supplementary material for: Influence of hypomagnetic field on the heartbeat in zebrafish embryos
Source: Front Physiol. 2022 Oct 21;13:1040083. doi: 10.3389/fphys.2022.1040083 (PMC9634549; doi:10.3389/fphys.2022.1040083)
Supplement: Supplementary file 6 [file Image1.pdf]

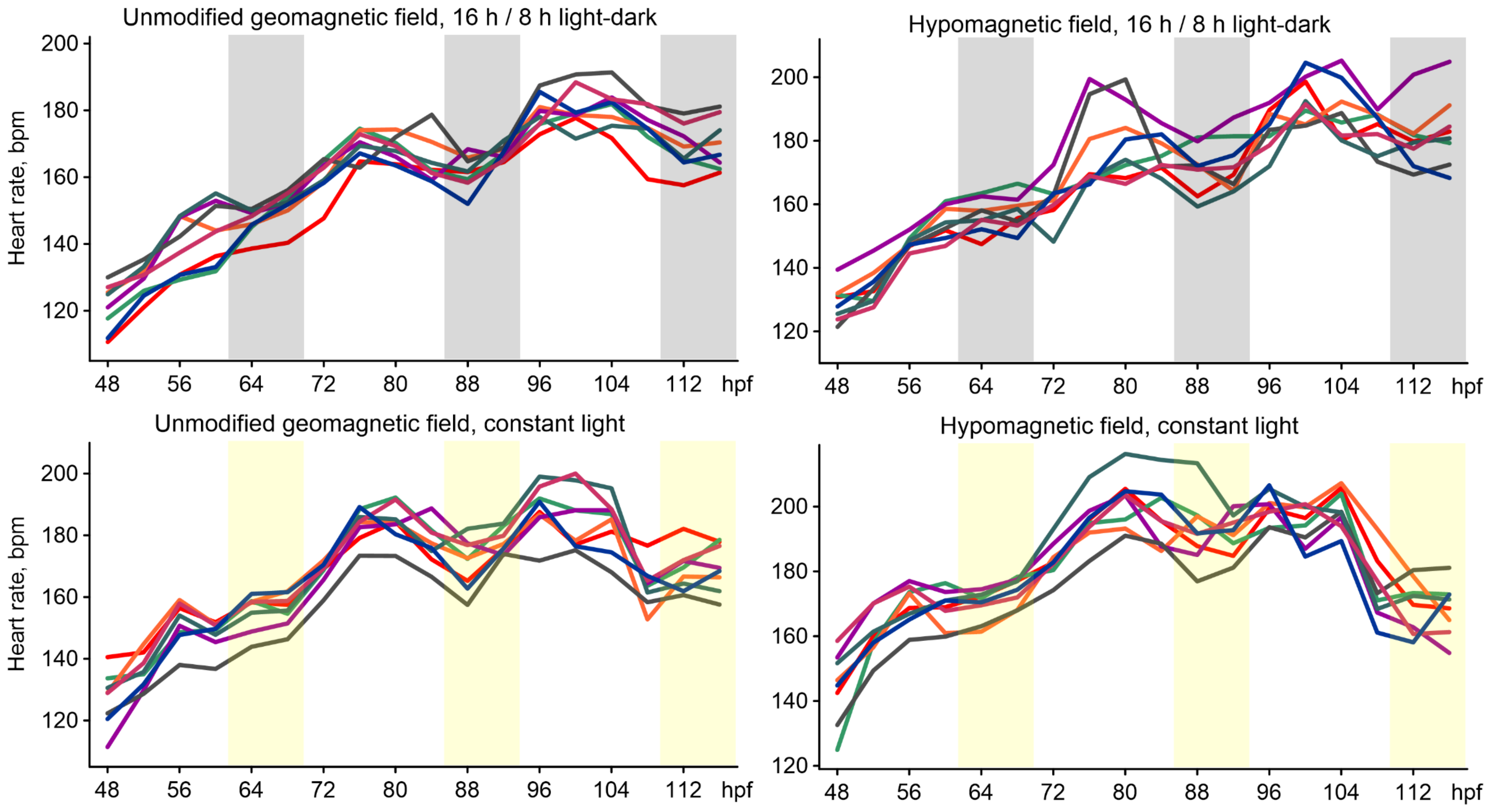

Fig. Suppl. 1. Heartbeat rate in zebrafish individuals exposed to HMF and the geomagnetic field. Gray bars on the above graphs represent the dark phase, and the light bars on the below graph are the expected dark phase under constant illumination.
